# A Molecular Dynamics Study of Ag-Ni Nanometric Multilayers: Thermal Behavior and Stability

**DOI:** 10.3390/nano13142134

**Published:** 2023-07-23

**Authors:** Florence Baras, Olivier Politano, Yuwei Li, Vladyslav Turlo

**Affiliations:** 1 ICB, UMR 6303 CNRS-Université de Bourgogne, 9 Avenue A. Savary, 47870 Dijon, France; olivier.politano@u-bourgogne.fr (O.P.); yuwei.li@laas.fr (Y.L.); 2 Empa, Swiss Federal Laboratories for Materials Science and Technology, Laboratory for Advanced Materials Processing, Feuerwerkerstrasse 39, 3602 Thun, Switzerland; vladyslav.turlo@empa.ch

**Keywords:** nanometric metallic multilayers, immiscible metals, Ag–Ni interface, thermal stability, grain boundary groove, molecular dynamics simulations

## Abstract

Nanometric multilayers composed of immiscible Ag and Ni metals were investigated by means of molecular dynamics simulations. The semi-coherent interface between Ag and Ni was examined at low temperatures by analyzing in-plane strain and defect formation. The relaxation of the interface under annealing conditions was also considered. With increasing temperature, a greater number of atomic planes participated in the interface, resulting in enhanced mobility of Ag and Ni atoms, as well as partial dissolution of Ni within the amorphous Ag. To mimic polycrystalline layers with staggered grains, a system with a triple junction between a silver single layer and two grains of nickel was examined. At high temperatures (900 K and 1000 K), the study demonstrated grain boundary grooving. The respective roles of Ni and Ag mobilities in the first steps of grooving dynamics were established. At 1100 K, a temperature close but still below the melting point of Ag, the Ag layer underwent a transition to an amorphous/premelt state, with Ni grains rearranging themselves in contact with the amorphous layer.

## 1. Introduction

Nowadays, thin films are utilized in various advanced technologies, such as protective coatings (diffusion or thermal barriers, wear or corrosion protection), biosensors, plasmonic devices, optical coatings, electrical operating coatings, and thin-film photovoltaic cells. Among these, nanometric metallic multilayers (N2Ms), also known as layered composites or nanolaminates, represent a promising new type of thin film. N2Ms consist of hundreds of nanometric layers of two metallic elements, each layer being a few nanometers thick (ranging from 4 to 100 nm) and composed of nanometric columnar grains. Compared to monolithic films, N2Ms exhibit outstanding properties due to the numerous interfaces associated with their nanoscale layered microstructure. They have garnered considerable attention thanks to their unique physical and chemical properties [1].

In the present work, we will focus on a specific type of N2Ms composed of immiscible metals, which we will refer to as N2MIs. There are numerous possible compositions of N2MIs, each with unique properties that make them of interest to researchers. However, the thermal stability of N2MIs remains a significant concern, as thermal annealing would result in a dramatic change in their layered microstructure, therefore limiting their potential applications [2]. Experimental studies on the lack of structural stability in N2MIs during heat treatment have been conducted. Some examples include Cu/Ag [3,4], Cu/W [5,6], Cu/Mo [7], Hf-Ti [8], Cu-Ag/Fe [9], Ag/Ni [10,11], etc.

**Figure 1:**
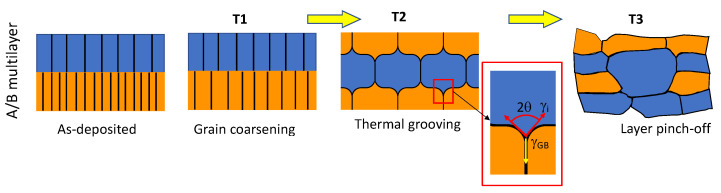
Schematic representation of the degradation of a N2MI A/B as a function of temperature. The inset represents enlarged view of the triple junction.

When submitted to annealing, N2MIs are known to degrade via grain coarsening, grain boundary grooving, and pinch-off of the layers, as illustrated in Figure 1. The intersection of a grain boundary and an interface leads to the formation of a triple junction (see the inset in Figure 1). The groove angle between the two interfaces is given by the equilibrium condition:

(1) $$2\cos\left(\theta \right)=\gamma_{\mathrm{GB}}/\gamma_{i}$$

where $\gamma_{\mathrm{GB}}$ is the grain boundary energy and $\gamma_{i}$ the interface energy. Equation (1) assumes that the energy of the two interfaces around the triple junction is equal. Once the groove angle is established, the groove gradually extends, eventually penetrating through the entire grain thickness. However, despite this observation, there remains a significant knowledge gap regarding the fundamental mechanisms involved, particularly the diffusion processes occurring at interfaces and grain boundaries that contribute to the alterations in the microstructure.

To investigate the degradation of the initial laminated architecture, we employed molecular dynamics simulations (MD). This technique is particularly suitable for modeling the thermal stability of N2Ms, as it enables analysis at the nanoscale, which is the typical length scale of these systems. Our aim is to gain valuable insights into the underlying mechanisms that occur at the microscopic level and understand the influence of several factors, including lattice mismatch, local stresses, interface orientation, creation of defects, mobility, and atomic diffusion.

In recent years, microscopic simulations have played a pivotal role in studying reactive metallic nanometric multilayers, such as Al-Ni or Al-Ti nanofoils, to elucidate the fundamental mechanisms responsible for their enhanced reactivity (for a comprehensive review, refer to Baras et al. [12], and the relevant references therein). The self-propagating exothermic reaction in Al-Ni N2Ms was first investigated at the nanoscale using MD simulations in [13]. The propagation of the reaction front has also been studied in Al-Ni nano-laminated composites with a complex structure, as reported in [14,15]. Different microstructures, namely amorphous, single crystal, columnar grains, and randomly oriented grains of varying size, were considered to evaluate their influence on propagation characteristics [16]. Moreover, the role of grain boundaries on diffusion during nanolaminate combustion was evaluated in [17]. In the present study, the emphasis is on N2Ms composed of immiscible metals. In theory, these metals are considered non-reactive, suggesting that nanolaminates should display stability. However, experimental evidence contradicts this notion when they undergo heat treatment. Hence, it is imperative to understand the impact of the nanolaminate structure on their behavior during the annealing process, with particular attention given to their behavior at grain boundaries.

The phenomenon of grain boundary grooving has been investigated by means of MD simulations in various situations. For example, Iwasaki and Miura [18] examined this phenomenon in Al- and Cu-supported films to assess the role of the substrate in adhesion. Nam and Srolovitz [19] investigated the penetration of liquid Ga into Al bicrystals to assess the possibility of intergranular fracture under negligible stress. Shibuta et al. [20] conducted large-scale MD simulations to examine the evolution of grain boundary grooves during the solidification of iron in relation to the characteristics of the grain boundary. However, the grain boundary grooving phenomenon in N2MIs has yet to be investigated using MD simulations.

As a case study, we will focus our attention on the thermal stability of the Ag/Ni nanolaminated system. The stability of Ag-Ni laminates has been evaluated in [2,10,11] with evidence of grooving under annealing at high temperatures. On the other hand, Ag-Ni nanometric multilayers have been investigated for their exceptional mechanical properties [21] and in the context of radiation tolerance applications [22]. This particular system can be considered an example of nanometric multilayers composed of immiscible metals with semi-coherent interfaces. The objective of our work is to investigate the microscopic-scale mechanisms that contribute to the lack of stability of these systems at high temperatures.

To begin our investigation, we started with a simple bilayer system (Ag-Ni) that models the N2MI structure by stacking one layer of Ag between two layers of Ni. In the Ag/Ni system, both elements have the same face-centered cubic lattice, with the misfit ranging from 10% to 20%. This results in a semi-coherent interface, which we studied in terms of residual in-plane strain and misfit dislocation emergence due to interface stress relaxation. Moving forward, we considered a bilayer system (Ni-Ag) with two grains of Ni in the corresponding layer to investigate the influence of a columnar microstructure on the grain boundary grooving process. By analyzing this system, we can gain further insight into the factors that affect the thermal stability of the Ag/Ni nanolaminate system.

## 2. Methods

The simulations were performed using Large-Scale Atomic/Molecular Massively Parallel Simulator (LAMMPS) software [23], and the Embedded Atom Method (EAM) potential, developed by Pan et al. for the Ag-Ni binary system [24]. The structural parameters (lattice parameter and cohesive energy) and thermodynamic properties (melting temperature) are reported in Table 1. The calculated values of melting temperature are close to the experimental ones. The temperature evolution of the lattice parameter $a_{0}$ was evaluated and results are reported in Appendix A.

Bimetallic interfaces can be classified according to their crystal structure and mismatch between the two lattices [1]. At coherent interfaces, two metals in contact have identical crystal structures with similar values of lattice parameters. At semi-coherent interfaces, the two metals have the same crystal structures but the lattice misfit is larger than 10% and smaller than 20%. In order to quantify the lattice mismatch, it is usual to introduce the lattice misfit defined as

(2) $$f=\frac{a_{0}\left(A\right)}{a_{0}\left(B\right)}$$

where $a_{0}\left(A\right)$ and $a_{0}\left(B\right)$ are the lattice parameters of metals A and B. In the case of the layered Ag-Ni system, the interface can be considered as a semi-coherent interface since

(3) $$f=\frac{a_{0}\left(\mathrm{Ag}\right)}{a_{0}\left(\mathrm{Ni}\right)}=1.1826$$

The misfit at interface induces a *strain* that can be accommodated by the system. The temperature dependence of the misfit can be easily evaluated given the thermal expansion of each metal (see Appendix A).

**Figure 2:**
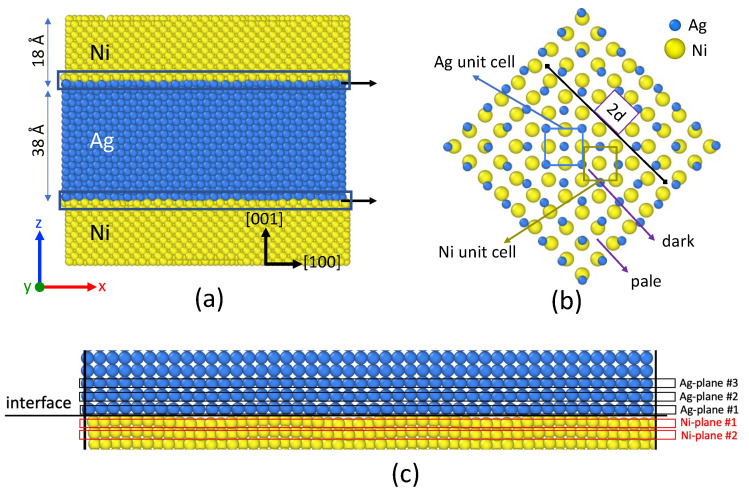
(**a**) Initial configuration of the system A, with one Ag slice in between two Ni layers. The Ag and Ni atoms are shown as blue and yellow spheres. The typical size of the simulation box is $L_{x}=L_{y}=9.5$ nm and $L_{z}=8.1$ nm. (**b**) Snapshot of the interface with one atomic layer of Ag and one atomic layer of Ni. The smaller blue spheres correspond to the Ag atoms. The distance between two pale zones is 2d. (**c**) Enlarged front view of the interface. Numbering of the Ag and Ni atomic planes around the interface.

Two representative atomic configurations were prepared, with a different microstructure (see Table 2). Typically, we designed system A as a bilayer Ni-Ag with semi-coherent (001) interfaces, by stacking two slabs of 22 atomic planes thick for Ag and 26 for Ni and displacing the atoms along the *z* direction through periodic boundaries by half the thickness of the Ni slab, as shown in Figure 2a. Along the lateral directions, the simulation box contained 46 layers of Ag and 54 layers of Ni. This specific choice corresponds to a superlattice (23 × a(Ag)/27 × a(Ni)∼1) that minimizes the global strain in the *x* and *y* directions. The system is composed of 53,636 atoms (20,102 Ag and 33,534 Ni atoms). The explicit numbering of atomic planes around the interfaces is given in Figure 2c.

It is well-known that superimposing two periodic patterns leads to Moiré fringes associated with pale and dark zones (superimposition or opposition of the lattice lines). In 1D systems, the distance between pale and dark zones, *d*, is

(4) $$2d=\frac{p^{2}}{\delta p}+p$$

where *p* and p+δp are the periods of the two 1D lattices. The atoms arrangement at the (001) interface is displayed in Figure 2b, in the case of the bilayer Ag-Ni system. As expected, we observed pale and dark zones, typical of Moiré fringes. In dark area, we observed Ag unit cells in the Ag layer and Ni unit cells in the Ni layer. We expect that atoms keep their *fcc* structure in dark zones.

**Figure 3:**
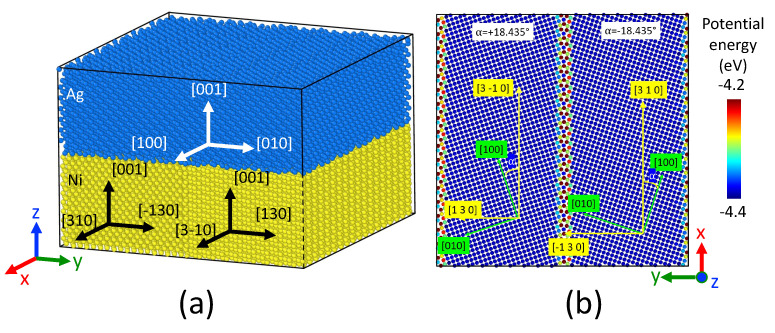
(**a**) Initial configuration of system B, with one Ag layer (18,432 atoms) and one Ni layer (32,400 atoms). The Ni layer is composed of two grains with a tilt angle of 36.8 deg around [001] axis. The Ag and Ni atoms are shown as blue and yellow spheres. The typical size of the simulation box is $L_{x}=L_{y}=10.1$ nm and $L_{z}=6.9$ nm. (**b**) Snapshot of one atomic layer of Ni, perpendicular to the *z* axis.

For system B, we considered a bilayer system with two grains in the Ni layer, as shown in Figure 3a. The atomic configuration corresponds to a Σ5(310)[001] symmetric tilt grain boundary that was built using Atomsk software [26] by rotating the two lattices by an opposite angle of magnitude $18.435^{\circ }$ (Figure 3b). The corresponding grain boundary energy was evaluated in [27] for the Pan potential: $\gamma_{\mathrm{GB}}=1.38$ J/m${}^{2}$, which is close to the DFT estimation $\gamma_{\mathrm{GB}}=1.26$ J/m${}^{2}$ [28]. This system is designed to investigate grain boundary grooving near the melting point of Ag, at which extreme grain growth is expected in the Ag layer, thus allowing us to approximate such layer with a single crystal. At the same time, the melting point of Ag corresponds to around 0.7 melting point of Ni, at which considerable grain growth is also expected to occur, however, preserving the significant amount of grain boundaries in the Ni layer. By considering symmetric tilt grain boundaries here, we ensure the equivalence of Ag/Ni interfaces within each grain, making such configuration thermally stable against grain growth. Thus, Ni grain boundary wetting and grooving can be characterized with atomic precision while maintaining the system size relatively small.

The thermal stability of the bilayer was then investigated. Periodic boundary conditions were applied in all directions. The equations of motion were integrated with a time step equal to 1 fs. At each temperature, the system was equilibrated in the NPT (constant number of atoms, temperature, and pressure) ensemble using Nose-Hoover thermostat and Parrinello-Rahman barostat for at least 1 ns. The damping parameters were 0.1 ps for the thermostat and 1.0 ps for the barostat. Several temperatures were considered in this study, while zero pressure was maintained in all directions. Before analyzing the configurations, a brief minimization without changing box dimensions is performed to eliminate thermal fluctuations in the atomic positions.

The atomic positions were visualized with OVITO [29]. The local atomic environment (i.e., *fcc*, *hcp*, or *unk*, representing face-centered cubic, hexagonal close-packed, and unknown crystal structures, respectively) was determined using polyhedral template matching (PTM) using a RMSD cutoff equal to 0.1 (default value). Later on, we refer to the atoms with defective, disordered, or just different than *fcc* or *hcp* crystal structures simply as unknown atoms. In addition, the interfaces were characterized with different indicators, for example, potential energy and atomic volume given by the Voronoï volume. The Voronoï tessellation of the simulation box is performed by taking each atoms as Voronoï cell centers. Each cell defines the volume region occupied by the corresponding atom and is a good approximation of the local atomic volume. Dislocation line defects in the crystal were determined using the DXA analysis in OVITO [30].

## 3. Results

### 3.1. System A: Analysis at Low Temperature (20 K)

We first analyzed system A at a low temperature (20 K) after relaxation for 1 ns.

**Figure 4:**
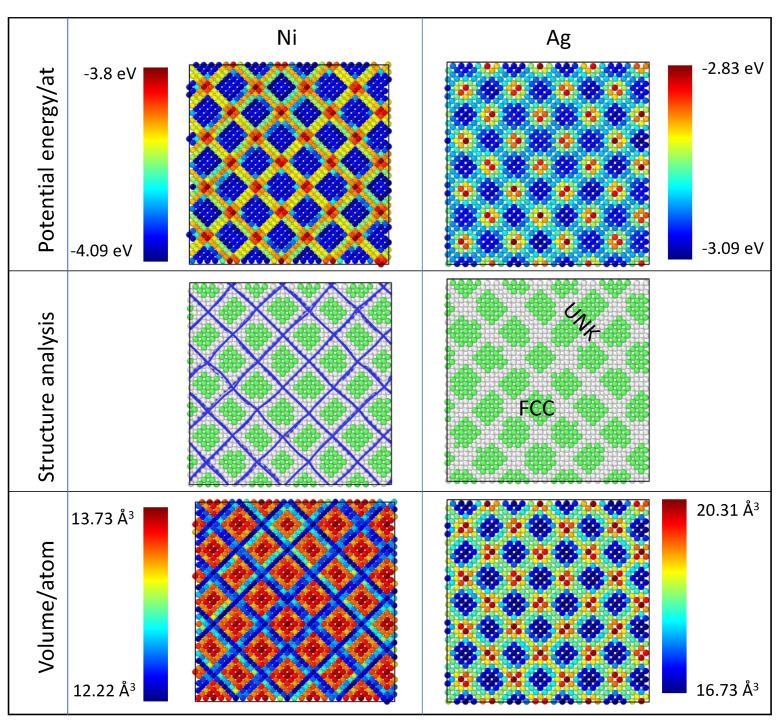
Snapshots of the interface at low temperature (20 K) after 1 ns relaxation. Left: Ag plane #1; Right: Ni plane #1. Atoms are colored according to their atomic potential energy, local atomic crystal structure, and Voronoï atomic volume. Each color bar gives the range of variation in the corresponding quantity. For the structure analysis, green atoms are *fcc*, and gray ones are detected as *unk*. Blue lines are the interfacial misfit dislocation lines plotted on the top of the Ni plane #1 for visualization purposes.

Figure 4 displays the snapshots of the Ag and Ni planes #1, adjacent to the interface. Atoms were colored according to different indicators. We recovered the typical Moiré fringes observed in Figure 2b. The crystallographic structure proves that atoms in the dark zones are detected as *fcc*, whereas atoms in pale zones are considered as *unk* by the PTM analysis. The dislocation analysis gives dislocation lines along the defected zones at interfaces. The potential energy per atom is lower for *fcc* atoms than for *unk* atoms. Atoms in pale areas are less stable (ΔE=0.26 eV for Ag and 0.29 eV for Ni), especially for atoms at crossing fringes. As reported in Table 1, the reference value of the energy/atom for the bulk atom is $E_{0}^{\mathrm{Ni}}=-4.385\;\;\mathrm{eV}$, lower than for Ni atoms at interfaces. In the case of Ag, the reference value $E_{0}^{\mathrm{Ag}}=-2.97\;\;\mathrm{eV}$ is in the range of interface atoms energy.

The snapshots with the atomic volume/atom also reflect the Moiré patterns. In the Ag plane, the *fcc* atoms in dark zones occupy a smaller volume than those in pale zones. In the case of *fcc*-Ag atoms, the atomic volume of bulk atoms (the bulk value refers here to a quantity evaluated in the middle of each layer) is $V=a_{0}{\left(\mathrm{Ag}\right)}^{3}/4=18$ Å${}^{3}$, larger than the Voronoï volume of *fcc*-Ag atoms at the interface (16.7 Å${}^{3}$). In the case of a *fcc*-Ni atoms, the atomic volume of bulk atoms is $V=a_{0}{\left(\mathrm{Ni}\right)}^{3}/4=10.9$ Å${}^{3}$, smaller than *fcc*-Ni atoms at interface. This quantity is the signature of the different in-plane strain states of Ag and Ni atoms at interfaces: *fcc*-Ag atoms are in compression, while *fcc*-Ni atoms are in tension.

**Figure 5:**
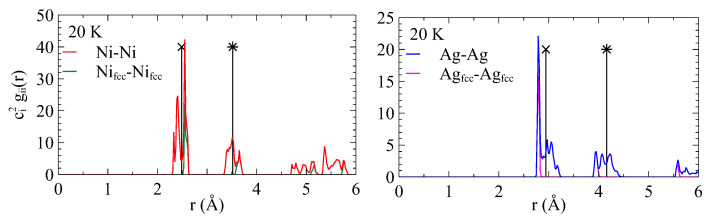
Partial radial distribution function $g_{ij}\left(r\right)$ where *i* and *j* stand for Ag and Ni, and *r* is the distance between atoms. The radial distribution function reads $g\left(r\right)=c_{i}^{2}\;g_{ii}\left(r\right)+2c_{i}c_{j}\;g_{ij}\left(r\right)+c_{j}^{2}\;g_{jj}\left(r\right)$, where $c_{i}$ and $c_{j}$ are the concentrations of *i* and *j*. The function was calculated for Ni plane #1 and Ag plane #1 at the interface at 20 K. Vertical lines with a cross indicate the distance between the first neighbors in the bulk system (inside the layers). Vertical lines with a star are located at the bulk lattice parameter (second neighbors). Ni${}_{\mathrm{fcc}}$ refers to *fcc*-Ni atoms and Ag${}_{\mathrm{fcc}}$ to *fcc*-Ag atoms.

The radial distribution functions calculated for the atoms located in the interfacial planes only (see Figure 5) corroborates this observation. In bulk *fcc* systems, the first peak is located at $r_{1}=a_{0}/\sqrt{2}$. At interfaces, several peaks appear from either side of this value. In the Ag plane, the peak to the left precisely corresponds to *fcc*-Ag atoms. This indicates that *fcc*-Ag atoms at interfaces are closer to each other than in bulk. The in-plane strain on *fcc*-Ag atoms due to lattice mismatch reads

(5) $$\epsilon_{\mathrm{fcc}-\mathrm{Ag}}=\frac{r_{1,i}-r_{1,b}}{r_{1,b}}=-0.051$$

where $r_{1,i}$ is the location of the *fcc* peak at interface and $r_{1,b}$ is the bulk value. In the Ni layer, the peak to the right precisely corresponds to *fcc*-Ni atoms. The in-plane strain on *fcc*-Ni atoms due to lattice mismatch reads

(6) $$\epsilon_{\mathrm{fcc}-\mathrm{Ni}}=\frac{r_{1,i}-r_{1,b}}{r_{1,b}}=+0.037$$

It is important to note that such residual in-plane strain in *fcc*-Ag and *fcc*-Ni is one order of magnitude larger than the mismatch between Ag and Ni layers (0.0067) and, thus, the result of additional relaxation enabled near interface by misfit dislocation network.

**Figure 6:**
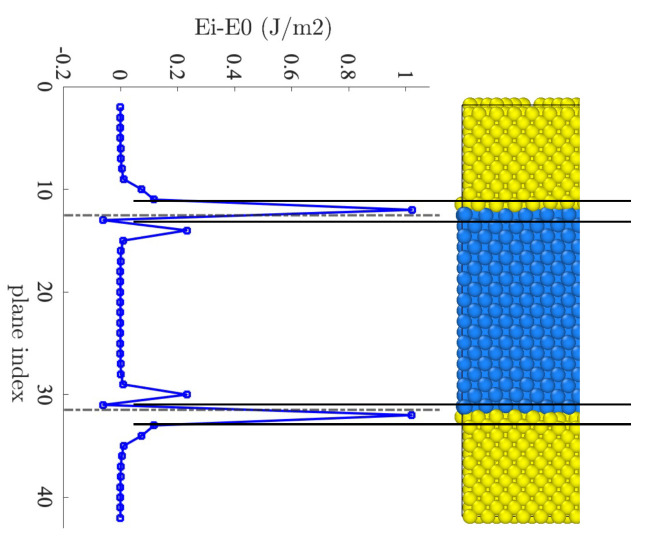
Excess of potential energy at the interface at 20 K.

The interfacial region can also be clearly identified according to the average potential energy of atoms in each plane adjacent to the interface after relaxation. Figure 6 depicts the excess of potential energy compared to bulk energy in each atomic plane parallel to the interface. The potential energies of the planes near the interface significantly differ from the bulk region (i.e., region far away from the interface in the middle of the layer). The interface energy, $\gamma_{i}$, reads:

(7) $$\gamma_{i}=\frac{1}{A}\;\left[\sum_{\ell }\left(E_{\ell }^{\mathrm{Ni}}-E_{0,\ell }^{\mathrm{Ni}}\right)+\left(E_{\ell }^{\mathrm{Ag}}-E_{0,\ell }^{\mathrm{Ag}}\right)\right]$$

where $E_{\ell }$ is the actual potential energy of the plane *ℓ*, $E_{0,\ell }=N_{\ell }^{\mathrm{Ni}}E_{0}^{\mathrm{Ni}}+N_{\ell }^{\mathrm{Ag}}E_{0}^{\mathrm{Ag}}$ is the reference bulk energy, with $N_{\ell }^{\mathrm{Ni}}$ the number of Ni in the plane *ℓ*, $N_{\ell }^{\mathrm{Ag}}$ the number of Ag in the plane *ℓ*, and *A* is the interface area. The interface energy evaluated with Equation (7) is $\gamma_{i}=1.39\;\;{J/m}^{2}$ for an interface with the Ag(001)/Ni(001) orientation, which is on the upper bound of interface energies predicted with different interatomic potential (1.1–1.4 J/m${}^{2}$) [31]. While theoretical and experimental data for Ag(001)/Ni(001) interfaces is absent in the literature, lower values were reported in the literature for other out-of-plane misorientations, ranging on average from 0.7 to 0.9 J/m${}^{2}$ [32,33]. Such low values can be caused by lower energy (111) orientation of at least one metal [32], or they can be caused by the theoretically predicted decrease in interface energy with increasing temperature (see here [34], for example) as experimental measurements correspond to high temperatures near Ag melting point [33]. In any case, by selecting a high-energy Ag(001)/Ni(001) interface in this work, we ensure enhanced interfacial mobility that allows us to investigate the early stages of thermal degradation and grain boundary grooving on MD timescales.

### 3.2. System A: Analysis at High Temperatures

To explore the temperature sensitivity of the system, we conducted MD simulations at various temperatures, below the melting point of Ag. Initially, we focused on the system’s behavior during short timescales (1 ns) to gain insights into its relaxation mechanism at the interface. Subsequently, we extended our analysis to longer timescales (up to 60 or 100 ns) to assess its stability under high-temperature conditions (900 K and 1100 K).

**Figure 7:**
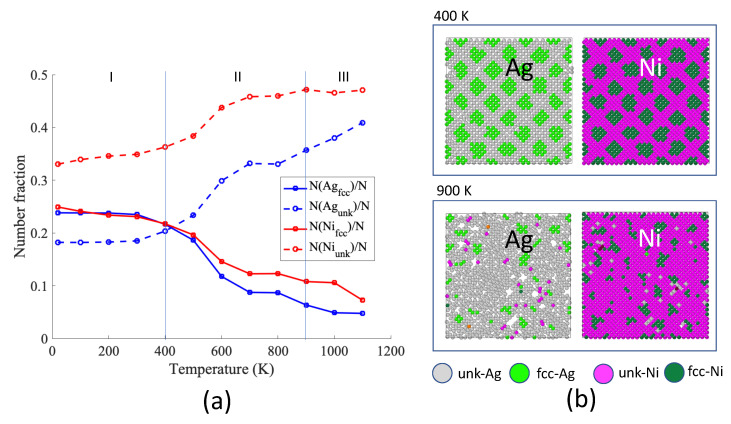
(**a**) Number fraction of *fcc* and *unk* atoms at interfaces, as a function of temperature. *N* is the total number of atoms in the first planes of Ag and Ni at interface (planes #1). (**b**) Snapshots of Ag and Ni planes #1 at the interface at 400 K and 900 K, at 1 ns. Atoms are colored according to their chemical identity (Ag or Ni) and their local structure (*fcc*, *unk*).

The temperature dependence of the number fraction of *fcc* and unknown atoms at interfaces (first Ag and Ni planes) is depicted in Figure 7. We noticed three regions:

- Region I from 20 K to 400 K: $N\left({\mathrm{Ag}}_{\mathrm{fcc}}\right)=N\left({\mathrm{Ni}}_{\mathrm{fcc}}\right)$, other atoms (unknown) are located at Moiré fringes;
- Region II from 500 K to 800 K: $N\left({\mathrm{Ag}}_{\mathrm{fcc}}\right)\leq N\left({\mathrm{Ni}}_{\mathrm{fcc}}\right)$, more Ag atoms lose their *fcc* structure than Ni ones when increasing the temperature;
- Region III from 900 K to 1100 K: $N({\mathrm{Ag}}_{\mathrm{fcc}})$ and $N({\mathrm{Ni}}_{\mathrm{fcc}})$ further decrease and most of the interface is composed of unknown atoms.

At low temperatures (region I), the number of *unk*-Ni is larger than the number of *unk*-Ag because the initial interface is composed of more Ni than Ag atoms. At intermediate temperatures (region II), the number of *fcc*-Ag decreased more than the number of *fcc*-Ni. This indicates that the Ag layer is more sensitive to temperature due to its lower melting point.

**Figure 8:**
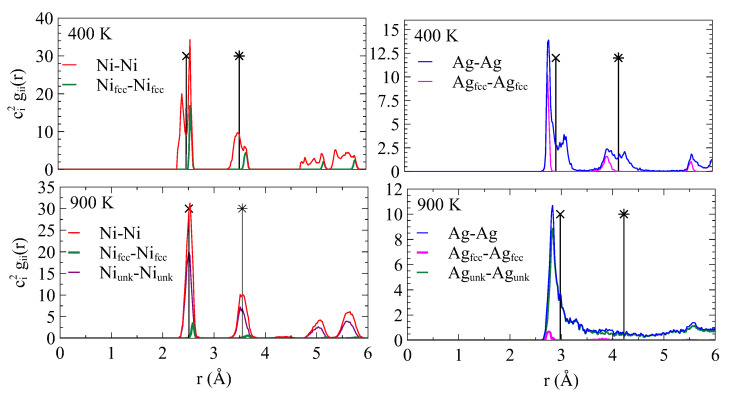
Partial radial distribution function $g_{ij}\left(r\right)$ where *i* and *j* stand for Ag and Ni, and *r* is the distance between atoms. The radial distribution function reads $g\left(r\right)=c_{i}^{2}\;g_{ii}\left(r\right)+2c_{i}c_{j}\;g_{ij}\left(r\right)+c_{j}^{2}\;g_{jj}\left(r\right)$ where $c_{i}$ and $c_{j}$ are the concentrations of *i* and *j*. The function was calculated for the Ni plane #1 and Ag plane #1 at the interface at 400 K and 900 K. Vertical lines with a cross indicate the distance between the first neighbors in the bulk system (inside the layers). Vertical lines with a star are located at the bulk lattice parameter (second neighbors).

Figure 7b shows the snapshots of Ag and Ni planes #1 at interfaces. At 400 K, we still observe the typical Moiré pattern for the two planes. The situation is completely different at 900 K where the Ag plane is mostly composed of unknown atoms with some vacancies. Very few clusters of *fcc*-Ag persist. The same observation can be deduced from the partial radial distribution function of Figure 8. At 400 K, the partial radial distribution function demonstrates the typical behavior observed at low temperatures: the first peak is split into two parts for Ag and Ni. Because the first peak to the left of the reference value corresponds to *fcc*-Ag, *fcc*-Ag atoms are in compression. Because the second peak to the right of the reference value corresponds to *fcc*-Ni, *fcc*-Ni atoms are in tension.

At a temperature of 900 K, the first peak of the radial distribution function $g_{\mathrm{AgAg}}$ noticeably broadened, while the second peak vanished, indicating a loss of ordering. The first silver plane at the interface (Ag plane #1) transformed into an amorphous state at this temperature. In contrast, for $g_{\mathrm{NiNi}}$, the split peaks narrowed, and distinct peaks remained indicating long-range order. The first nickel (Ni) plane at the interface (Ni plane #1) preserved its ordered structure. On the other hand, the plane-by-plane organization persists.

**Figure 9:**
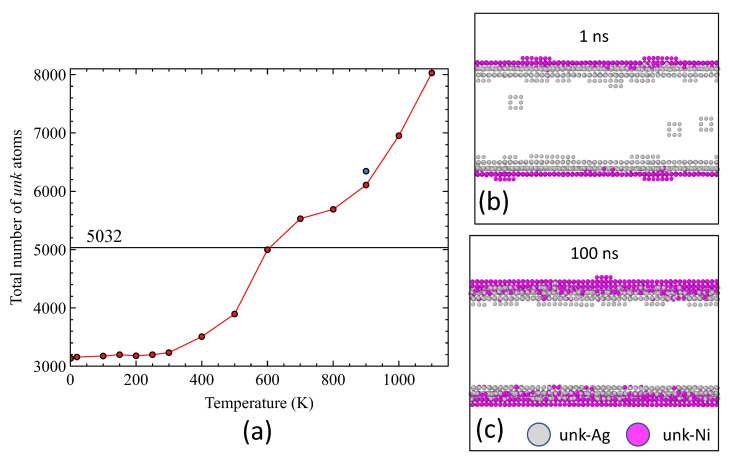
(**a**) Total number of unknown atoms in the system as a function of temperature after 1 ns relaxation. The blue point is the number of unknown atoms at 900 K after 100 ns. (**b**) Front view of the system at 900 K in which only unknown atoms are selected at 1 ns, (**c**) at 100 ns.

Figure 9a gives the total number of *unk* atoms as a function of temperature after 1 ns. If we assume that the interface is composed of one Ag plane and one Ni plane, the number of atoms at interfaces is 5032. If the total number of *unk* atoms exceeds this value, that means that there are *unk* atoms located outside the strict location of the interface. The front view of the system at 900 K (Figure 9b) demonstrates that several Ag planes, in the inner Ag layer, became occupied by *unk* atoms. This corresponds to the amorphization of Ag planes close to the interface, at temperatures well below the melting point of Ag. That observation is consistent with the RDF behavior (see Figure 8). At 900 K, the number of *unk* atoms after 100 ns slightly exceeds (<4%) the number of *unk* atoms detected after 1 ns. It is expected that the disordering at interfaces promotes mobility (see Section 3.3). At high temperatures, the lattice misfit is accommodated in 1 ns and this relaxation is followed by an effective mixing between *unk*-Ni and *unk*-Ag atoms, as shown in Figure 9c.

**Figure 10:**
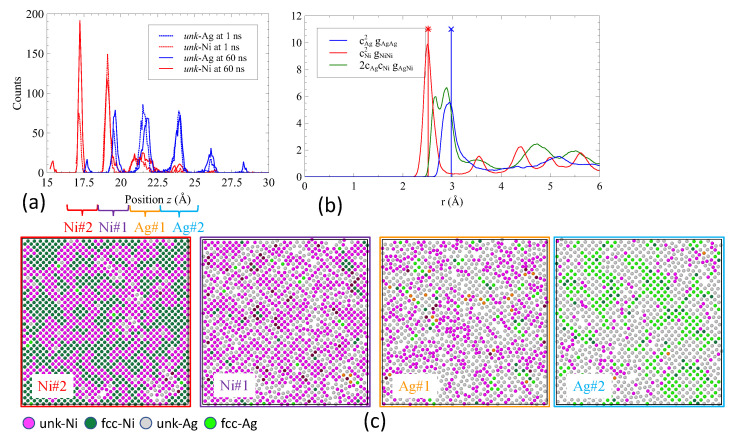
(**a**) Histogram of the *z* position of all unknown atoms detected at 1100 K (lower interface) at 1 ns and 60 ns. (**b**) Partial radial distribution functions $g_{ij}\left(r\right)$ where *i* and *j* stand for Ag and Ni, and *r* is the distance between atoms. The radial distribution function reads $g\left(r\right)=c_{\mathrm{Ag}}^{2}\;g_{\mathrm{AgAg}}\left(r\right)+2c_{\mathrm{Ag}}c_{\mathrm{Ni}}\;g_{\mathrm{AgNi}}\left(r\right)+c_{\mathrm{Ni}}^{2}\;g_{\mathrm{NiNi}}\left(r\right)$ where $c_{\mathrm{Ag}}$ and $c_{\mathrm{Ni}}$ are the concentrations of Ag and Ni. The function was calculated for all *unk* atoms at 1100 K after 60 ns. Vertical lines indicate the distance between the first neighbors in the bulk system (inside the layers) for Ni and Ag. (**c**) Top view of atomic planes detected in the histogram at 1100 K. Atoms are labeled according to their species (Ag and Ni) and structure (*fcc*, *unk*, and *hcp*).

Figure 10a illustrates the histogram of the *z* position of *unk* atoms at 1100 K, after 1 ns and 60 ns. Clear peaks corresponding to atomic planes were observed. After 1 ns, the mixing was limited to the first two planes (Ni plane #1 and Ag plane #1) at interface (We kept the plane numbering shown in Figure 2c although atoms are exchanged between planes). However, after 60 ns, the mixing became more efficient. The first plane of Ni atoms from the interface contained *unk*-Ag atoms, while the second plane primarily consisted of *unk*-Ni atoms. The population of *unk*-Ag atoms was spread across four planes. Furthermore, the first Ag plane (#1) from the interface contained a significant amount of *unk*-Ni atoms. The second Ag plane (#2) contained just a few *unk*-Ni atoms. The peaks in the histogram appear to be broadened, indicating a partial amorphization accompanied by mixing of Ni and Ag atoms.

Additionally, in Figure 10b, the partial radial distribution function demonstrated that Ni atoms maintained a crystallographic ordering, while Ag atoms lost long-range ordering. The cross radial distribution function $g_{\mathrm{AgNi}}\left(r\right)$ exhibited a split first peak and showed a tendency for ordering at long distances. The snapshots corresponding to the atomic planes are depicted in Figure 10c. In the Ni layer, planes #1 and #2 exhibit clear evidence of local ordering, where outgoing Ni atoms are replaced by incoming Ag atoms. On the other hand, Ag plane #1 in the Ag layer shows a random distribution of Ag and Ni atoms. In Ag plane #2, there is a mixture of *fcc*-Ag and *unk*-Ag, with some *unk*-Ni atoms distributed within the amorphous zones. The exchange of atoms in the four atomic planes leads to the formation of vacancies that promote in-plane mobility.

### 3.3. System A: Mobility at Interfaces

The mobility of the atoms was evaluated with the mean squared displacement (MSD) that measures the position *r* of the atoms with respect to a reference position $r_{0}$ over time:

(8) $$MSD=<{\left(r\left(t\right)-r_{0}\right)}^{2}>=2nDt$$

where *n* is the dimension of the system and *D* is the diffusion coefficient.

**Figure 11:**
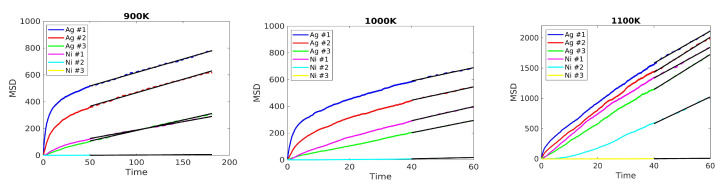
MSD as a function of time for atoms located initially in planes #*i* at 900 K (**left**), 1000 K (**middle**), and 1100 K (**right**). Dashed lines indicate the linear range where the diffusion coefficients were computed.

We performed simulations over a long time, at least 60 ns, and measured the MSD for atoms that were initially located in five planes (three Ag planes and two Ni planes) around the two interfaces at t=0. The MSD as a function of time is plotted in Figure 11. We noted that Ag atoms in Ag plane #1 are more mobile than Ag atoms in Ag plane #2. However, after some time, there is an exchange of atoms from plane #1 and plane #2. For both planes, the MSD grows at a similar rate in the long time limit. Atoms in Ni planes are less mobile than Ag atoms. Only Ni atoms at interfaces (plane #1) move.

**Figure 12:**
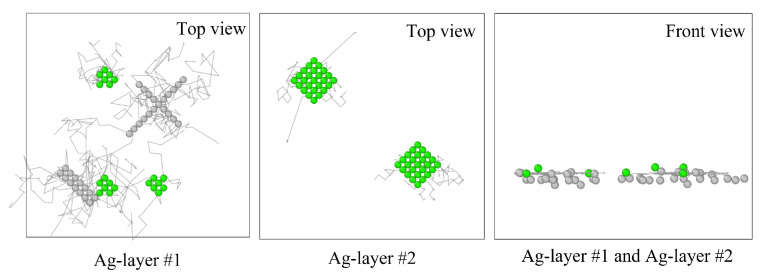
Trajectories of selected atoms at 900 K over 10 ns. Gray (*unk*) and green (*fcc*) dots indicate the initial position of the diffusing atoms.

The trajectory analysis was also used to observe the movement of atoms as shown in Figure 12. We selected *fcc* atoms and *unk* atoms in Ag plane #1 and Ag plane # 2 and the lines indicate the trajectory of atoms from beginning to the end (i.e., 10 ns). From Ag plane #1, the *unk* atoms show more mobility compared with *fcc* atoms, which can also be seen in Ag plane #2, and that most of the *fcc* atoms stay at a confined position. Thus, we can conclude that atomic displacements occur mainly along misfit dislocations. The comparison between the top view and the front view reveals the predominance of displacements in the *x* and *y* directions.

Table 3 gives the diffusion coefficients (see Equation (8)) for atoms that were initially in the different planes. Note that an atom moving to another plane still contributes to the calculation of the diffusion coefficient. At a temperature of 900 K, the diffusion coefficients for Ag planes #1 and #2 exhibit similar values, while the diffusion coefficient for Ni plane #1 is half that of Ag atoms. Moreover, the diffusion coefficient for Ni plane #2 approaches zero. When the temperature increases to 1000 K, the diffusion coefficient for Ni plane #1 becomes comparable to that of Ag planes #1, #2, and #3. At a temperature of 1100 K, the diffusion coefficients experience a significant increase compared to lower temperatures. Activation energies are also reported in Table 3.

For comparison, we developed separate MD simulations to evaluate the coefficient of self-diffusion of Ag in liquid and undercooled liquid in the temperature range [900 K–1800 K]: $D_{\mathrm{liq}}^{\mathrm{Ag}}=5.84\times 10^{-9}\;\exp\left(-E_{a}/k_{B}T\right)$, with $E_{a}=0.335$ eV. Its value at 1000 K, $D_{\mathrm{liq}}^{\mathrm{Ag}}\left(1000K\right)=1.19\times 10^{-9}$ m${}^{2}$/s, is significantly larger than the value measured at the interface (see Appendix B). This clearly indicates the amorphization of Ag at the interface without premelting.

### 3.4. System B: Thermal Stability

**Figure 13:**
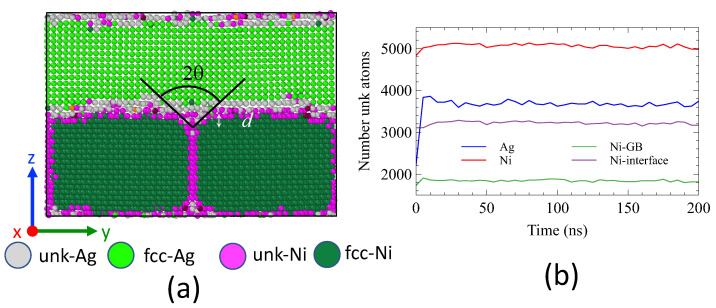
(**a**) Snapshot of the system after 200 ns at 900 K. Only atoms in a slice of 10 Å in the *x* direction are shown. Atoms are colored according to their species (Ag and Ni) and structure (*fcc*, *unk*). (**b**) Number of unknown atoms as a function of time.

We then proceeded to study System B, which consists of one layer of Ag and two grains of Ni, as illustrated in Figure 3, at various temperatures below the melting point of Ag. The simulations were conducted over a duration of 200 ns. Figure 13 depicts the system at a temperature of 900 K, where we observed a slight rounding of the grains with a depth *d* not exceeding 4 Å (approximately the lattice constant $a_{0}$ of Ni). The evolution of the number of unknown atoms is presented in Figure 13b. After a brief relaxation time (<10 ns), the number of *unk*-Ag in each interface, reached a maximum before stabilizing at a constant value (1850), which exceeds the number of atoms in an atomic plane of Ag (1152). The number of *unk*-Ni, which corresponds to one atomic plane, remained constant throughout the simulation. The grooving angle $2\theta ∼95^{\circ }$ between the grains is difficult to estimate due to the small *d* value.

**Figure 14:**
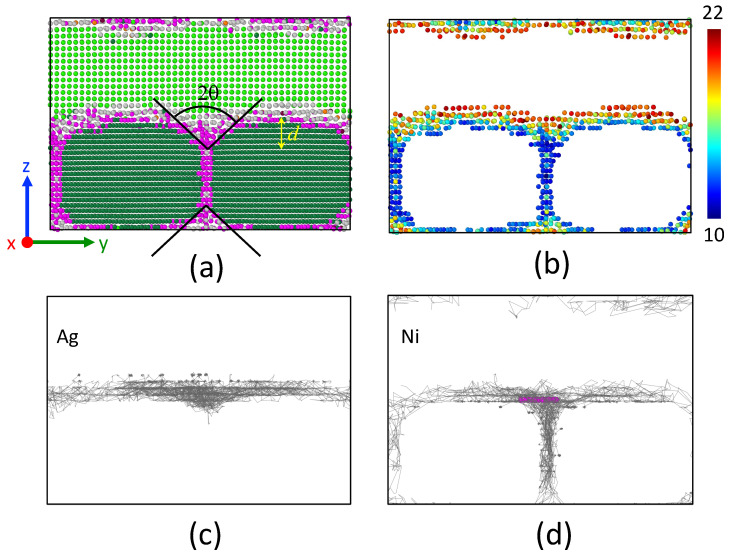
(**a**) Snapshot of the system at 1000 K. Only atoms in a slice of 10 Å in the *x* direction are shown. Atoms are colored according to their species (Ag and Ni) and structure (*fcc*, *unk*) as in Figure 13. (**b**) Volume per atom of *unk* atoms. The color bar indicates the limits: 10 Å${}^{3}$ and 22 Å${}^{3}$. (**c**) Trajectories of selected Ag atoms (20) that were initially at the interface. (**d**) Trajectories of selected Ni atoms (20) that were initially at the interface.

The system snapshot at 1000 K after 200 ns is presented in Figure 14a. Our observations show a spheroidization of the Ni grains as compared to Figure 13a. An amorphous region composed of *unk*-Ni and *unk*-Ag atoms surrounds the *fcc* Ni grains. The grooving depth, approximately d∼9 Å, and the grooving angle, $2\theta ∼95^{\circ }$, are clearly noticeable.

The temperature dependence of the groove angle is intimately linked with the variation of grain boundary and interface energy with temperature (Equation (1)). However, estimating this variation can be challenging, particularly at high temperatures where enthalpy and entropy contributions must be considered. In the MD simulations, Equation (1) shows that at temperatures of 900 and 1000 K, the grain boundary energy ($\gamma_{\mathrm{GB}}$) is 1.35 times larger than the interface energy ($\gamma_{i}$).

A crucial issue is to understand the mechanisms responsible for grooving. For this purpose, we first characterized the nature of the interface by evaluating the number of *unk*-Ni and *unk*-Ag atoms. The population of *unk*-Ag rapidly increased to 4000 (t<10 ns) and then stabilized around 4250. This indicates that two atomic planes of Ag in each of the interfaces have become amorphous. In contrast, the population of *unk*-Ni at the interfaces remained around 3500, which corresponds to one atomic plane of Ni becoming amorphous. At interfaces, the population of Ag atoms exceeds that of Ni by 20%. This is not surprising because Ag has the lowest melting point.

The amorphous nature of the interface promoted atom mobility. The trajectories of 20 selected Ag and Ni atoms, which were located initially at interfaces, are depicted in Figure 14c,d. Trajectories of Ag atoms are limited to the interface itself and the grain boundary groove region. In contrast, Ni atoms moved along the interface and in the grain boundaries. Their trajectories run along the rounded solid grains. The passage in the grain boundary is difficult for the atoms of Ag because their atomic volume (i.e., metallic radius) is greater than that of the atoms of Ni (see Figure 14b). In addition, the solubility limit for Ag in Ni grain boundaries might be also restricted by relatively low free volume at the grain boundaries, thus providing the low thermodynamic driving force for Ag diffusion.

The mean square displacements along the *y* and *z* directions were calculated for Ag and for Ni atoms. As an example, the MSD along *y* of Ag atoms is defined as

(9) $${\mathrm{MSD}}_{\mathrm{Ag}}^{y}\left(t\right)=\sum_{i=1}^{N_{\mathrm{Ag}}}{\left(y\left(t\right)-y_{0}\right)}^{2}/N_{\mathrm{Ag}}$$

where $N_{\mathrm{Ag}}$ is the total number of Ag atoms. This quantity reveals the amplitude of displacements in each direction, depending on the species. Silver atoms exhibit efficient movement in the *y* direction, while displacements of Ag atoms in the *z* direction are nearly negligible. Conversely, for Ni atoms, the mean squared displacement, ${\mathrm{MSD}}_{\mathrm{Ni}}^{y}$, along the *y* direction is greater than that along the *z* direction, ${\mathrm{MSD}}_{\mathrm{Ni}}^{z}$. This indicates more effective diffusion at interfaces in comparison to grain boundaries.

**Figure 15:**
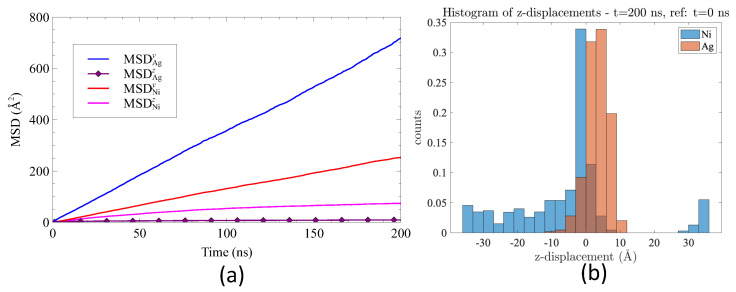
(**a**) Mean square displacements (MSD) along the *y* and *z* directions for Ag and Ni atoms (all atoms were considered) as a function of time. (**b**) Histogram of displacements along the *z* direction of atoms located in planes Ag#1 and Ni#1. The displacement of atoms is calculated at t=200 ns with a constant reference frame at t=0 ns.

We calculated the displacements of atoms initially located in Ag and Ni planes #1 along the *z*-direction over the time span of 0 to 200 ns. The resulting histogram is depicted in Figure 15b. The displacements of Ni atoms exhibit a wide range of values, indicating that Ni atoms from Ni plane #1 are traversing the grain boundary, moving from one interface to the other. Conversely, Ag atoms from Ag plane #1 predominantly remain near the interface, close to their original positions along the *z* axis. The histogram also provides a rough estimate of the interface thickness, which is approximately 15 Å. This interface corresponds to the thickness over which both Ni and Ag atoms move.

**Figure 16:**
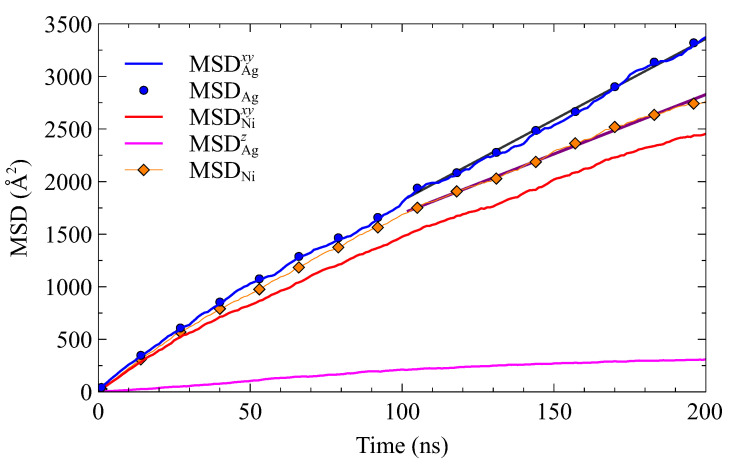
Mean square displacements of Ag and Ni atoms located at the interface (first planes Ag#1 and Ni#1) as a function of time. Solid lines represent the slopes calculated using linear regression over the time interval [100,200]. Time units is ns. Temperature is 1000 K.

Following the methodology outlined in Section 3.3, we calculated the mean square displacements (MSDs) of two sets of atoms in Ag and Ni planes #1 at a temperature of 1000 K (see Figure 16). The MSD values obtained in the *x* and *y* directions provide insights into the mobility parallel to the interface. Conversely, the MSD in the *z* direction reflects the mobility perpendicular to the interface, particularly across the grain boundary. For comparison, MSDs in the three directions (Equation (8) with n=3) were also computed.

The diffusion coefficients (Equation (8)) were estimated in the interval 100–200 ns. Their values are reported in Table 4.

Values of *D* are significantly larger than those estimated for System A at 1000 K (see Table 3). The presence of a triple junction and interface misorientation seem to promote mobility. The mobility of Ag atoms is greater than that of Ni atoms. However, Ag mobility is mostly limited to planes parallel to the interface. The mobility of Ni atoms takes into account interface and grain boundary diffusion. Grain boundary diffusion can be evaluated with MSD${}_{\mathrm{Ni}}^{z}$. The corresponding diffusion coefficient value $D_{z}$ is five times smaller than $D_{xy}$.

**Figure 17:**
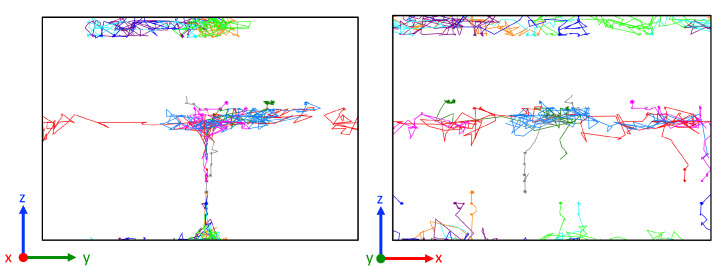
Trajectories of 10 selected Ag atoms located in the middle grain boundary over 200 ns. Segments represent atom displacement over 1 ns. Each color corresponds to a single Ag atom. **Left**: front view of the boundary. **Right**: lateral view of the grain boundary plane.

As depicted in Figure 14a, a few Ag atoms were detected within the cores of the Ni grain boundary. After 200 ns, the number of these atoms reached 26. Additionally, a larger number of Ag atoms became trapped within the funnel, delineating the groove of the grain boundary. The trajectories of ten representative Ag atoms, which are trapped within the grain boundary at the middle of the Ni layer, are illustrated in Figure 17. Initially, these Ag atoms moved along the interface before reaching the grain boundary towards the end of the simulation. They penetrated the grain boundary at various locations along the *x* direction. Throughout the simulation, no Ag atom was observed to cross the grain boundary.

**Figure 18:**
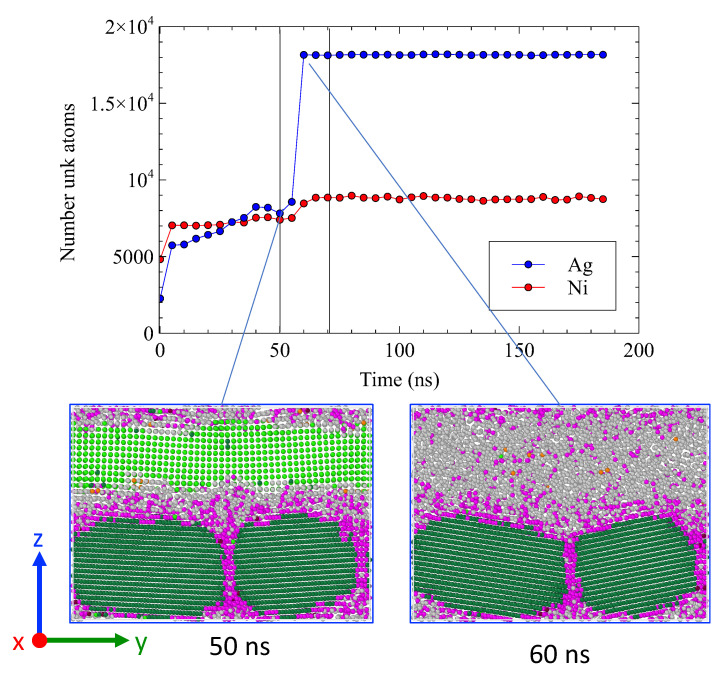
Evolution of the number of *unk* atoms as a function of time. Snapshots of the system at 50 and 60 ns. Atoms are colored according to their type and structure.

The system evolution at 1100 K is illustrated in Figure 18. Within the first 10 ns, the number of *unk* atoms increased rapidly. Subsequently, the number of *unk*-Ni remained stable around 7000, corresponding to approximately 1.5 atomic planes at each interface. In contrast, the number of *unk*-Ag continued to rise from 6000 (equivalent to 2.6 atomic planes/interface) to 8000 (equivalent to 3.5 atomic planes/interface) at 55 ns. Throughout this stage, the number of Ni atoms dissolved in the amorphous layer remained constant.

Between 55 and 60 ns, the entire Ag layer underwent amorphization, as evident from the sudden increase in the *unk*-Ag population. This behavior exhibits characteristic features of a first-order phase transition, which can be attributed to the melting point depression commonly observed in nanomaterials [35,36]. Following the standard method, we calculated the diffusion coefficient after the complete amorphization of the Ag layer, yielding a value of $D=1.5\times 10^{-9}$ m${}^{2}$s${}^{-1}$. This value is in close proximity to the self-diffusion coefficient observed in liquid systems, which is $D=1.75\times 10^{-9}$ m${}^{2}$s${}^{-1}$ (see Appendix B).

**Figure 19:**
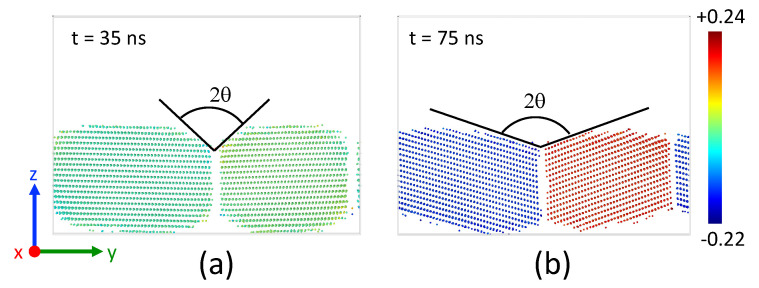
Snapshot of the system at 1100 K. Only *fcc*-Ni are shown. Atoms are colored according to their orientation along *x* calculated with the PTM analysis: (**a**) 35 ns, (**b**) 75 ns.

The snapshots presented in Figure 18 capture the structural changes taking place in the Ag layer before and after the transition. In Figure 19, only *fcc*-Ni atoms of the two solid grains are shown. Prior to the transition, both grains maintained their original orientation and became round-shaped with the same groove angle as that observed at lower temperatures. Remarkably, a groove depth, of about 10 Å, was formed in under 30 ns.

Following the transition, the situation changed significantly: the grain boundary started to move, and the two grains rearranged themselves with different orientations along *x*. The amorphous layer in which the grains are embedded allows for free reorientation. Faceting of the Ni grains was observed. The groove angle 2θ underwent a significant widening, from 95${}^{\circ }$ to 140${}^{\circ }$. This widening could indicate a relaxation in grain boundary energy. The grain boundary energy actually decreases from 1.92 J/m${}^{2}$ before the transition to 1.26 J/m${}^{2}$ after the transition.

**Figure 20:**
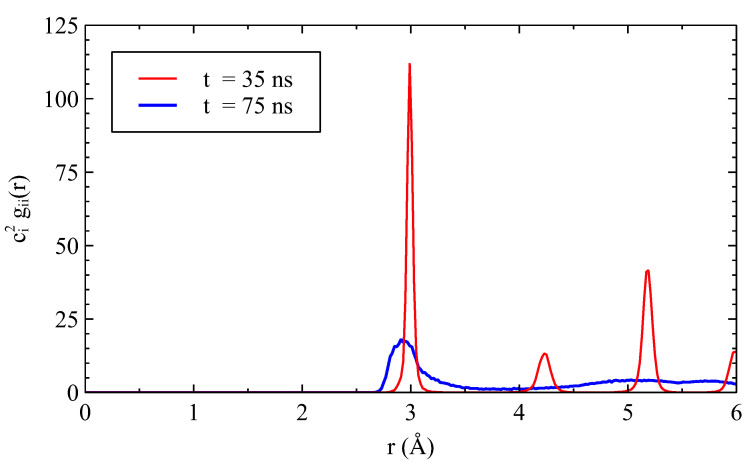
Radial distribution function of Ag atoms located in the slice in *z* [45,61] Å.

The structural change in the Ag layer is demonstrated in Figure 20. The secondary peaks of the pure Ag solid, clearly discernible at 35 ns, in a region in the middle of the Ag layer, disappeared at 70 ns. After the transition, the RDF is typical of that of an amorphous solid.

## 4. Discussion

By means of molecular dynamics simulations, we investigated the behavior of a nanometric Ag-Ni multilayer system. This system serves as a model for studying the relaxation of semi-coherent interfaces.

Initially, we focused on a bilayer configuration consisting of two monolayers (referred to as System A) and specifically examined a (001) interface. To minimize global strain, we employed a superlattice structure in the *x* and *y* directions and investigated the relaxation process of lattice mismatch at low temperatures. Distinct Moiré patterns emerged, characterized by dark areas composed of *fcc* atoms and pale areas occupied by unidentified and less stable atoms. At the interfaces, the *fcc* and *unk* atoms occupied different volumes, as evident from coordination analysis that revealed two discrete peaks deviating from the bulk value. In the Ag plane, the *fcc* atoms experienced compression, while, in the Ni plane, the *fcc* atoms experienced tension. Analyzing the interface energy, we found that the potential energy deviated from the reference value within a range of 5 atomic planes around the Ni-Ag interface at 20 K. The measured value of the interface energy was determined to be 1.39 J/m${}^{2}$, which was higher compared to other orientations.

Subsequently, the system was simulated at various temperatures to gain insight into the strain relaxation during annealing. The distinctive Moiré patterns remained visible up to 400 K. Beyond this temperature, a notable increase in the number of unknown atoms occurred within the first two Ag and Ni planes at the interface. When temperatures exceeded 700 K, the atoms in adjacent planes no longer retained their *fcc* structure. However, the system maintained a plane-by-plane arrangement. The atomic planes of silver underwent amorphization, while the nickel planes maintained a structure closely resembling the *fcc* arrangement, as confirmed by the in-plane radial distribution function. The interface thickness encompasses five atomic planes. It is worth noting that the relaxation occurred in less than 1 ns. Over a longer duration, we observed an exchange between Ni and Ag atoms, either through swapping or occupation of vacancies.

The trajectories of atoms at interfaces also reveal in-plane displacements at a short time and exchanges between adjacent planes (i.e., perpendicular to the interface) at longer times. This leads to an effective mixing at the interface between Ni and Ag that is unexpected for immiscible metals, accompanied with a local amorphization at interfaces, 200 degrees below the melting point of Ag. The mobility of atoms at interfaces is characterized by a diffusion coefficient of the order of magnitude of 10${}^{-11}$ m${}^{2}$/s, larger than the characteristic value in the *fcc* solid phase $10^{-15}$–$10^{-13}$ m${}^{2}$/s).

In the second part of the present work, we considered a system with a single layer of Ag above two grains of Ni. It is, in a way, the minimal system to account for more complex structures where polycrystalline layers are composed of columnar grains. System B is a section of a staggered system with a triple junction Ag/Ni-Ni. Other morphologies could be considered: a triple junction Ni/Ag-Ag or aligned grains. Although the constituents are immiscible metals, the layered structure may evolve upon annealing to pinched-off layers. Understanding the mechanisms leading to this phenomenon is a key issue.

In order to study the thermal grooving in Ag-Ni N2Ms, System B was simulated at high temperatures below the melting point of Ag. Three representative temperatures were considered: 900 K, 1000 K, and 1100 K.

At 900 K, we observed the formation of amorphous regions at the interfaces that are composed of more Ag than Ni atoms ($N_{\mathrm{Ag}}/N_{\mathrm{Ni}}=1.14$). Atoms of Ag planes #1 and #2, and Ni plane #1 composed this interfacial region. A widening of the triple junction is detectable with a grooving depth *d* that not exceeds two interplanar distances and a grooving angle $2\theta ∼95^{\circ }$.

At 1000 K, the interfacial amorphous region is thicker, with $N_{\mathrm{Ag}}/N_{\mathrm{Ni}}=1.2$. The atoms of the Ni plane #1 and Ag planes #1 and #2 constitute this amorphous region. The triple junction widened and a funnel formed above (below) the grain boundary. Thermal grooving was highlighted with a grooving depth *d* of five interplanar distances and a grooving angle $2\theta ∼95^{\circ }$. The amorphization of the interfacial region has two main consequences: the dissolution of *unk*-Ni in *unk*-Ag, together with an enhanced atomic mobility. Trajectories carried out by *unk*-Ni draw the grain contours, while trajectories of *unk*-Ag are limited to the interface (see Figure 14). The same observation is drawn by computing the displacements of atoms in the *z* direction: *z* displacements of *unk*-Ag do not exceed the interface limits (see Figure 15b). Silver atoms cannot rush into the grain boundary because their atomic volume is larger than that of Ni. Upon annealing, an induced dissolution is observed:

$${\mathrm{Ni}}_{\mathrm{sol}}+{\mathrm{Ag}}_{\mathrm{sol}}\to {\mathrm{Ni}}_{\mathrm{sol}}+{\left({\mathrm{Ni}}_{\mathrm{unk}}+{\mathrm{Ag}}_{\mathrm{unk}}\right)}_{\mathrm{interface}}+{\mathrm{Ag}}_{\mathrm{sol}}$$

**Figure 21:**
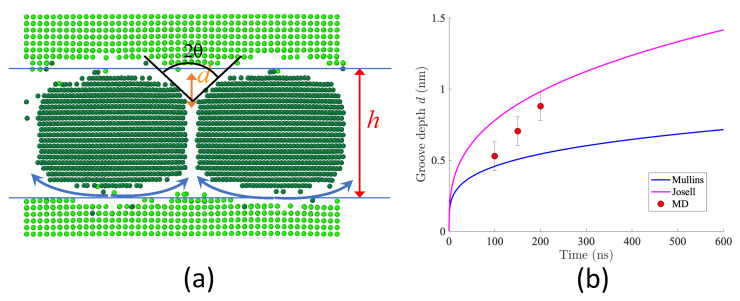
(**a**) The groove characteristics are visualized on a snapshot where only *fcc* atoms are shown: θ is the grooving angle, *d* the grooving depth, and *h* the layer thickness. The system box has been shifted to the center of the simulation box in order to see the two interfaces. (**b**) Grooving depth as a function of time is calculated with the Mullins and Shewnon theory (Equation (10)) and the Josel and Spaepen theory (Equation (11)).

The groove characteristics observed in System B after 200 ns of evolution are shown in Figure 21a. The system was visually centered to observe the two interfaces, revealing a distinctive rounding of Ni grains. Indeed, in the presence of a triple junction, the flat surface is not the equilibrium shape. The shape relaxation in poylcrystalline multilayers is associated with grain boundary grooving. In the present study, with a Ag <001>-grain sitting above two Ni <001>-grains, the dihedral angle 2θ is 95${}^{\circ }$. The study conducted by Lewis et al. [2] revealed a similar equilibrium groove angle of 99${}^{\circ }$ for a Ag <001>-grain sitting above two Ni <111>-grains. The obtained value signifies that the grain boundary energy is higher than the interface energy. Our results bolster the argument proposed by Lewis et al. that the layer with the highest grain boundary energy, typically the layer with the highest melting point, will be less stable in immiscible metal/metal systems.

One may question whether the equilibrium relation (1) that determines the dihedral angle is consistent with the estimation of interface and grain boundary energies. The estimation of the interface energy for System B with Equation (7) on either side of the grain boundary is nearly the same: $\gamma_{i-\mathrm{left}}=1.49$ Jm${}^{-2}$ and $\gamma_{i-\mathrm{right}}=1.48$ Jm${}^{-2}$. However, it evolves during the simulation and reaches 1.62 Jm${}^{-2}$. This is due to the contribution of a chemical term related to mixing (10%). The grain boundary energy significantly deviates from the reference value (1.38 Jm${}^{-2}$) and varies between 1.64 Jm${}^{-2}$ at 0 ns, 1.71 Jm${}^{-2}$ at 100 ns, and 2.0 Jm${}^{-2}$ at 200 ns. At 200 ns, the dihedral angle given by (1) is $\theta =51.9^{\circ }$, whereas the graphically measured value is 45${}^{\circ }$.

Another interesting aspect is to detect the kinetic path that leads to the shape relaxation we observed in MD simulations. Mullins developed a theory to describe the grooving in copper where the dominant transport mechanism is surface diffusion [37]. The diffusion equation associated with grain boundary grooving has been solved to obtain the kinetics of boundary grooving [38]. The expression was extended for a laminated system by substituting the interface free energy for the surface free energies:

(10) $$d=0.973\;\;\cos\theta \;\;{\left(\frac{D_{i}\gamma_{i}\nu \Omega^{2}}{k_{B}T}\;t\right)}^{1/4}$$

where $k_{B}$ is the Boltzmann constant. The parameters associated with System B are presented in Table 5. The diffusion coefficient, $D_{i}$, is determined based on the diffusion of Ni atoms at the interface ($D_{i}=D_{\mathrm{Ni}\#1}$). The interface energy, $\gamma_{i}$, and grain boundary energy, $\gamma_{\mathrm{GB}}$, are estimated using Equation (7) at 0 ns prior to the mixing of Ag and Ni. Furthermore, the atomic volume is estimated by considering the thermal expansion of both elements. The evolution of the grooving depth *d* given by Equation (10) and parameters of System B are depicted in Figure 21b.

Josell and Spaepen proposed a different expression for the grooving kinetics when the groove depth is of similar magnitude as the layer thickness [39]. In this case, the chemical potential gradient driving diffusion becomes established between adjacent boundaries and the time dependence of the groove depth reads [3]:

(11) $$d={\left(\frac{D_{i}\delta \;\gamma_{i}\Omega }{hk_{B}T}\;t\right)}^{1/3}$$

where *h* is the thickness of the Ni layer and δ the thickness of the diffuse interface. The corresponding grooving kinetics is plotted in Figure 21b for the properties reported in Table 5. The MD results fall within the range of the two theoretical expressions. The Josell and Spaepen estimation overestimated the observed groove depth of ∼ 8.24 Å.

At the higher temperature (1100 K), just below the melting point of Ag (1234 K), the amorphous interfacial region became thicker. The number of *unk*-Ag corresponds to 2.6 up to 3.5 atomic planes. This phenomenon led to a notable dissolution of Ni atoms from Ni planes #1 and #2. At 57 ns, a massive amorphization of the Ag layer occurred. Our observations are corroborated by the radial distribution function $g_{ii}$ of Ag atoms located in a crystallized slice at 35 ns as shown in Figure 20. The morphology of Ni grains exhibited a grooving effect until 55 ns with the same dihedral angle seen at lower temperatures and a groove depth of 10 Å. We also observed a reorganization of the Ni layer, with the slight displacement of the grain boundary. The morphology of the Ni grains then dramatically changed after 60 ns with a clear reorientation.

## 5. Conclusions

Our study represents an initial step towards comprehending the thermal stability of the Ag-Ni system at the atomic level and sheds light on the kinetic pathways accountable for the degradation of N2MIs. We have identified and emphasized two distinctive phenomena that are characteristic of N2MIs: grain boundary grooving and premelting. Molecular dynamics (MD) proves to be a suitable tool in this regard, as reliable embedded atom method (EAM) potentials exist to accurately describe the Ag-Ni binary system. The typical nanometric scales are directly accessible in MD. Moreover, MD allows direct access to typical nanometric scales. However, it is important to note that the time scales associated with the complete degradation of N2MIs may not align with those feasible in MD simulations. To overcome this limitation, we could utilize diffusive MD simulations, incorporating the diffusion coefficients estimated in the present study.

## Figures and Tables

**Table 1:** Parameters of pure metals computed with the EAM interatomic potential [24,25] and compared to experimental values. Lattice parameter and cohesive energy are evaluated at 0 K.

Parameter	Symbol	Ag (MD)	Ni (MD)	Ag (exp)	Ni (exp)
bulk melting temperature	$T_{m}$ (K)	1209	1728	1234	1728
lattice parameter	$a_{0}$ (nm)	0.416	0.352		
cohesive energy	$E_{0}$ (eV)	−2.97	−4.385		

**Table 2:** Characteristics of the two simulated systems. *N*(Ag) and *N*(Ni) represent the total number of Ag and Ni atoms.

System	$L_{x}=L_{y}$ (nm)	$L_{z}$ (nm)	*N*(Ag)	*N*(Ni)	Microstructure
A	9.5	8.1	18,432	20,102	Ni single crystal
B	10.1	6.9	32,400	33,534	Ni bicrystal

**Table 3:** Diffusion coefficients of each plane corresponding to the long time behavior.

Temperature	Ag #3	Ag #2	Ag #1	Ni #1	Ni #2	Units
900 K	0.26	0.34	0.34	0.21	0.007	$\times 10^{-11}m^{2}/s$
1000 K	0.77	0.83	0.83	0.88	0.075	
1100 K	4.84	4.85	4.42	4.25	3.69	
$E_{a}$	1.23	1.12	1.086	1.27	2.66	eV

**Table 4:** Diffusion coefficients of planes Ag#1 and Ni#1 in System B at 1000K.

	*D*	$D_{\mathrm{xy}}$	$D_{z}$	Units
Ag#1	2.60	3.89		$\times 10^{-11}m^{2}/s$
Ni#1	1.87	2.56	0.49	

**Table 5:** Properties of the Ag-Ni system (System B) evaluated at the target temperature. † The interface and grain boundary energies are evaluated at 0 ns.

		MD Value	Units
Temperature	T	1000	K
Interface diffusion coefficient of Ni	$D_{i}$	1.87 × 10${}^{-11}$	m${}^{2}$s${}^{-1}$
Interface energy ${}^{†}$	$\gamma_{i}$	1.49	Jm${}^{-2}$
Grain boundary energy ${}^{†}$	$\gamma_{\mathrm{GB}}$	1.64	Jm${}^{-2}$
Atomic volume	Ω	1.51 × 10${}^{-29}$	m${}^{3}$/at
Surface density (Number of atoms per surface area)	ν	1.35 × 10${}^{19}$	at/m${}^{2}$
Thickness of the Ni layer	*h*	3.5 10${}^{-9}$	m
Thickness of the diffuse interface	δ	0.5 10${}^{-9}$	m

**Figure A1:**
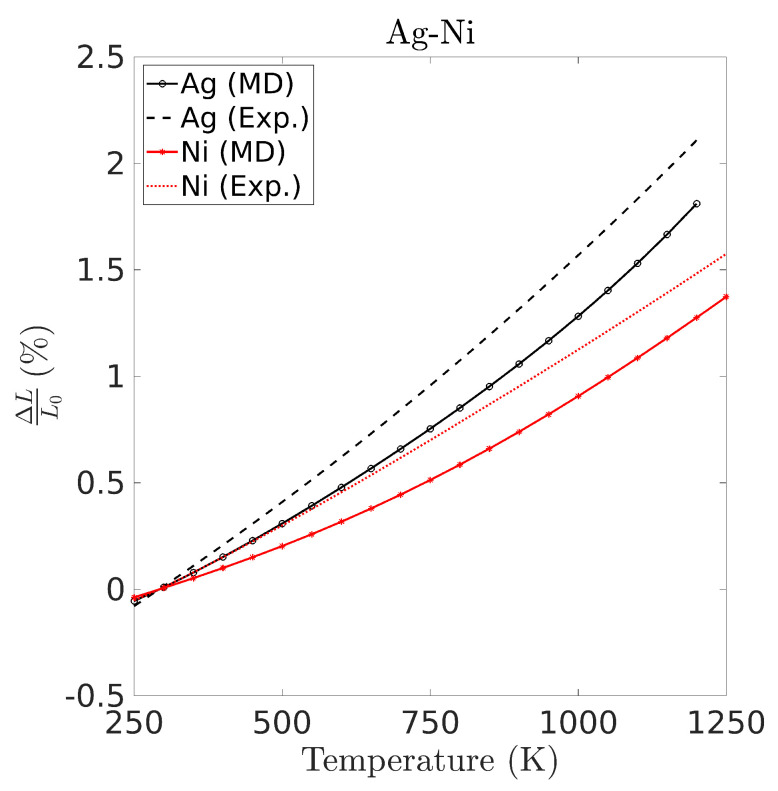
Linear expansion of the 2 elements. Comparison between MD results ans experimental values reported by Touloukian [40].

**Figure A2:**
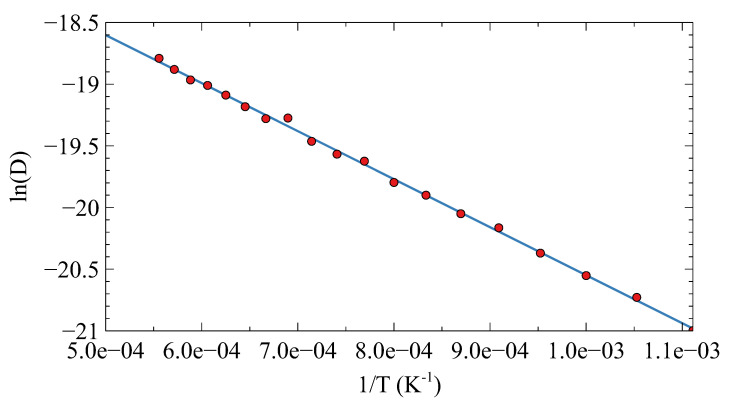
Logarithm of $D=D_{0}\;\exp\left(-E_{a}/kT\right)$ as a function of 1/T.

## Data Availability

The data presented in this study are available on request from the corresponding author.

## Appendix A. Linear Expansion

The thermal expansion coefficient was determined using a simulation box made of 10 × 10 × 10 lattice parameter. The initial temperature was set at 20 K and gradually increased to the temperature of interest in the NPT ensemble during 50 ps for Ag and Ni. To determine the lattice parameter at the target temperature, an additional 60 ps NPT run was performed at zero external pressure and the box size averaged during the last 10 ps. The thermal expansion coefficient $\alpha_{L}$ relates the change in temperature to the change in a material’s linear dimensions which can be expressed as:

(A1) $$\frac{▵L}{L_{0}}=\frac{a\left(T\right)-a_{0}}{a_{0}}=\alpha_{L}\cdot ▵T$$

where a(T) is the fitting curve of the lattice parameter and $a_{0}$ is defined as the lattice parameter at 293 K. This last expression was adopted by Touloukian [40], who published a collection of experimental values obtained for most of the elements present in the periodic table. For the two elements, MD results were fitted with a fourth-order polynomial (see Figure A1). The fitting coefficients A, B, C, D, and E ($a\left(T\right)=A+BT+CT^{2}+DT^{3}+ET^{4}$) are summarized in Table A1.

**Table A1:** Dependence of the lattice parameter $a\left(T\right)=A+BT+CT^{2}+DT^{3}+ET^{4}$ as a function of temperature.

	Ag	Ni
A	4.1605	3.5181
B	$1.7244\times 10^{-5}$	$3.1159\times 10^{-5}$
C	$9.2147\times 10^{-8}$	$-1.1012\times 10^{-8}$
D	$-7.5068\times 10^{-11}$	$3.0744\times 10^{-11}$
E	$3.0458\times 10^{-14}$	$-1.0023\times 10^{-14}$

## Appendix B. Diffusion Coefficient

The diffusion coefficient of silver was determined in the liquid and amorphous phases using specific simulations. Initially, a box containing a 6 × 6 × 6 fcc-Ag unit lattice (864 atoms) was set up. The atomic positions were randomized, and the system was then heated to 2000 K in the NPT ensemble for 1 ns to obtain a relaxed melt. Subsequently, the liquid metal was equilibrated at the desired temperature through a 0.5 ns run in the NPT ensemble. The box size was averaged during the final 0.1 ns. The mean-squared displacement was then accumulated over 1 ns in the NVT ensemble. An Arrhenius plot of atomic diffusivities computed with Equation (8) is presented in Figure A2). The linear fit gives $E_{a}=0.335$ eV and $D_{0}=5.84\times 10^{-8}$ (m/s${}^{2}$).
